# Complementary Metal–Oxide–Semiconductor-Based Magnetic and Optical Sensors for Life Science Applications

**DOI:** 10.3390/s24196264

**Published:** 2024-09-27

**Authors:** Tayebeh Azadmousavi, Ebrahim Ghafar-Zadeh

**Affiliations:** 1 Department of Electrical Engineering, University of Bonab, Bonab 5551395133, Iran; 2 Biologically Inspired Sensors and Actuators, Department of Electrical Engineering and Computer Science, Lassonde School of Engineering, York University, Toronto, ON M3J 1P3, Canada; 3 Department of Biology, Faculty of Science, York University, Toronto, ON M3J 1P3, Canada

**Keywords:** CMOS, optical sensors, magnetic sensors, PoC diagnostic

## Abstract

Optical and magnetic sensing methods are integral to both research and clinical applications in biological laboratories. The ongoing miniaturization of these sensors has paved the way for the development of point-of-care (PoC) diagnostics and handheld sensing devices, which are crucial for timely and efficient healthcare delivery. Among the various competing sensing and circuit technologies, CMOS (complementary metal–oxide–semiconductor) stands out due to its distinct cost-effectiveness, scalability, and high precision. By leveraging the inherent advantages of CMOS technology, recent developments in optical and magnetic biosensors have significantly advanced their application in life sciences, offering improved sensitivity, integration capabilities, and reduced power consumption. This paper provides a comprehensive review of recent advancements, focusing on innovations in CMOS-based optical and magnetic sensors and their transformative impact on biomedical research and diagnostics.

## 1. Introduction

**Figure 1:**
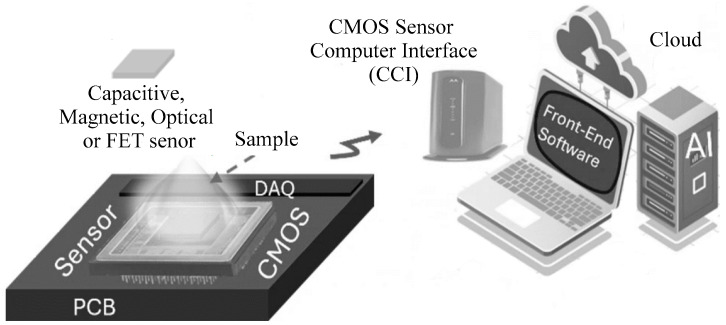
Illustration of a CMOS sensing platform consisting of a CMOS chip integrated with an electronic printed circuit board (PCB) for interfacing with a computer wirelessly. The data are transferred for AI cloud computation. The sensor can be magnetic, optical, or utilize other methods.

CMOS (complementary metal–oxide–semiconductor) sensors have significantly transformed the field of biosensing by enabling the integration of various sensing methodologies into a compact, cost-effective platform. The versatility of CMOS technology allows for the incorporation of multiple primary sensing methods, including field-effect transistors (FETs) [1,2,3], capacitive sensing [4,5,6,7], impedimetric sensing [8,9,10,11], optical sensing [12,13,14,15,16], and magnetic sensing [17,18,19,20,21,22,23], each contributing to a broad range of biomedical applications. A CMOS sensing platform consists of a sensing device connected to the interface circuit that reads the variation in the sensing device and converts the changes into a digital signal. All these components are integrated into a single CMOS chip. A data acquisition system is then used to transfer the information to a computer, with the collected data being transferred to a server or cloud for artificial intelligence (AI) analysis, as depicted in Figure 1.

FETs utilize the electric field effect to regulate the conductivity of a semiconductor channel, making them especially effective in biochemical sensing applications like deoxyribonucleic acid (DNA) sequencing. This capability has significantly contributed to the groundbreaking development of ion torrent technology, which integrates hundreds of millions of FET sensors [24,25]. Capacitive sensors, on the other hand, detect changes in capacitance caused by the interaction between the sensor and the target analyte—a method that is also useful for cellular monitoring and drug testing [26,27,28]—where the binding of nucleotides alters the capacitance. While the electrodes in capacitive sensors can be similar to those used in impedimetric sensors, the electronic interface circuitry between the two modalities differs, as reported in [8,10]. Magnetic and optical sensors respond to changes in magnetic or light properties, respectively, often using labels as described in sections II and III. It is noteworthy that the main methodology in this paper involves reviewing recent advances in optical and magnetic sensors developed through CMOS technology. Similarly, our team previously reported a paper on CMOS sensors focused on capacitive sensors [29].

One of the key advancements in the use of CMOS sensors is the integration of AI and machine learning (ML) for data analysis. AI and ML algorithms excel in pattern recognition, making them invaluable in processing the complex datasets generated by CMOS sensors. By analyzing vast amounts of data rapidly and accurately, these technologies enhance the sensitivity and specificity of biosensors, enabling the detection of subtle changes in biological systems. For example, in DNA sequencing, AI can identify mutations or patterns that might be missed by traditional analysis methods [30,31,32,33]. In cellular monitoring, ML algorithms can predict cellular behavior based on real-time data, allowing for early intervention in disease management [34,35,36,37]. Furthermore, the integration of AI and ML with CMOS sensors facilitates personalized medicine, as these technologies can tailor diagnostics and treatment plans based on individual patient data.

First, CMOS sensors are highly scalable and can be mass-produced at a relatively low cost thanks to established semiconductor manufacturing processes, making them accessible for widespread use [38,39,40]. Second, CMOS technology supports high-density integration, allowing multiple sensors to be incorporated into a single chip, thereby enhancing sensitivity and functionality [39,40,41,42,43,44,45]. Additionally, CMOS sensors exhibit low power consumption, which is critical for portable, wearable, and implantable medical devices [46,47,48,49,50,51,52].

This paper reviews recent advances in magnetic and optical CMOS sensors as described in Section 2 and Section 3, concluding with Section 4. These sections will provide a comprehensive overview of the state of the art in these specific sensor types, highlighting the innovations and potential applications that are driving progress in the field.

## 2. CMOS-Based Magnetic Sensors

Magnetic biosensors, with inherently non-magnetic biological samples, create low-background detection environments, obviating the need for sample pretreatment and ensuring matrix-insensitive measurements. In 1997, magnetic nanoparticles (MNPs) were initially employed as biomarker labels by the authors of [53], marking a significant advancement in biomolecular detection technologies. The MNPs, responsive to external magnetic fields, produce detectable stray fields, driving advancements in sensitivity, integration, and efficiency in biosensing applications. Thanks to the compatibility of magnetic biosensors with semiconductor-based fabrication processes, a compact and efficient sensing platform will be provided that offers portability and convenience and makes them ideal for point-of-care (PoC) diagnostics.

In recent developments, a variety of magnetic detectors have been introduced, presenting diverse operational mechanisms, including nuclear magnetic resonance (NMR), Hall effect, inductive, giant magnetoresistance (GMR), superconducting quantum interference device (SQUID), tunnel magnetoresistance (TMR), anisotropic magnetoresistance (AMR), and giant magnetoimpedance (GMI). While all of these sensors utilize MNPs to quantitatively detect analytes, their operational mechanisms vary significantly. The discussion of this section will primarily center on advancements in magnetic field sensors, particularly those harmoniously compatible with CMOS technology. Highlighting the progression in Hall effect, inductive, GMR, and NMR magnetic biosensors for biological applications, the focus lies on integrating these sensors through CMOS technology for PoC platforms.

### 2.1. Sensing Principle of Magnetic Sensors

**Figure 2:**
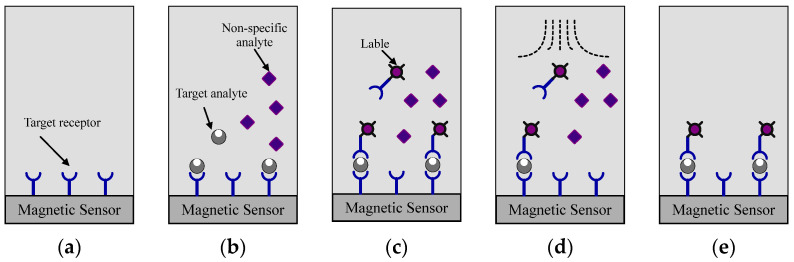
Magnetic sandwich immunoassay process: (**a**) the sensor surface is functionalized with a capture antibody, (**b**) the surface is then exposed to the sample, (**c**) a magnetically labeled detection antibody is introduced, (**d**) any non-specifically bound labels are washed away, (**e**) counting the remaining labels yields a measurement of the target concentration in the sample [17].

The process of MNP labeling by transforming biological data into identifiable signals via various assay setups plays a crucial role in magnetic sensing. These include clustering assay, direct labeling assay, magnetic amplification assay, and sandwich assay. Predominantly, detection methods involve a version of the sandwich immunoassay, illustrated in Figure 2 [17]. A target receptor is affixed to the surface of a magnetically responsive sensor. Subsequently, a sample is introduced into the assay. Following this, the magnetically labeled detection antibody is introduced. After non-specifically bound labels are washed away, the concentration of the target in the sample can be achieved through the generated magnetic field. Each of the magnetic sensors can convert the magnetic field generated by the MNPs linked to the target into an electrical readable signal in a unique way, which is briefly described through the following depiction.

### 2.2. CMOS-Based Interface Circuit of Magnetic Sensors

As previously noted, a variety of magnetic sensors have been engineered to detect magnetic signals from biological samples that are tagged with MNPs. This section will explore CMOS implementation of the interface circuit of magnetic sensors including inductive, Hall effect, GMR, and NMR sensors.

#### 2.2.1. Inductive Sensors

**Figure 3:**
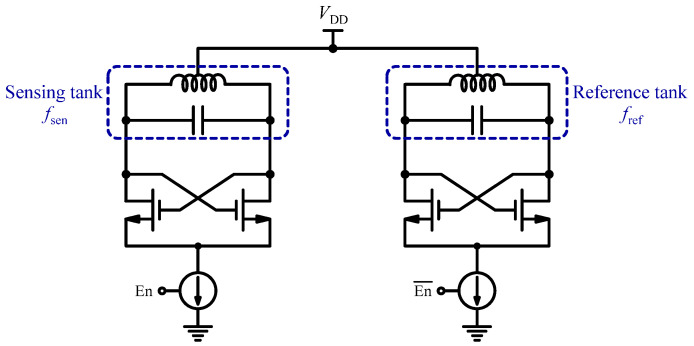
Differential magnetic sensing based on LC cross-coupled oscillator.

Inductive sensors operate on the fundamental principle of electromagnetic induction, which involves the detection of magnetic field variations through changes in inductance. In other words, the presence of targets alters the inductance of the sensor and consequently enables the detection and quantification of the biological entities of interest. For example, in [54], the authors developed a CMOS-based inductive sensor that employs an LC cross-coupled oscillator to detect target molecules labeled with magnetic beads. The frequency of the oscillator (1 GHz) changes in response to the presence of DNA samples. In this research that was implemented in a 0.13 µm CMOS process, differential sensing was introduced as shown in Figure 3. This method pairs a sensing oscillator with a reference oscillator, effectively reducing common-mode drift that may arise from variations in factors like supply voltage and temperature. The design incorporates eight sensor arrays that allow for parallel testing. Additionally, to enhance the delivery of samples, a microfluidic structure constructed from poly dimethylsiloxane (PDMS) was fabricated and securely bonded to the CMOS sensor chip. In another design by the authors of [55] for an LC cross-coupled inductive sensor, a correlated double-counting (CDC) configuration is utilized to enhance sensitivity, achieving an impressive 6 dB reduction in noise. This sensor features an array of 64 elements, implemented in a 65 nm CMOS, and it has been evaluated using DynaBeads^®^ MyOne™ Carboxylic Acid samples. The authors demonstrated the capability of real-time monitoring of the periodic and autonomous beating of progenitor cells through an integrated on-chip magnetic sensor. In another study of LC cross-coupled inductive sensors [56], the authors focused on sensing inductor design. This new design enhances the spatial uniformity of the transducer gain throughout the sensing area, thereby directly boosting the overall dynamic range of the system. In another study, the authors of [57] developed an LC cross-couple inductive microarray biochip for detecting clinical biomarkers in human samples that facilitate accurate disease diagnosis. It proposes a methodology for designing an on-chip inductor with a high-quality factor, compact size, and uniform magnetic field distribution. A microarray biochip with 14 sensor units was fabricated in a 0.18 µm RF CMOS process. Experimental results demonstrate a detection limit of 50 pg/mL (2.17 pM) and a dynamic detection range of 60 dB, showcasing the biochip’s effectiveness for clinical applications.

Recent advancements in cell-based sensing have introduced a groundbreaking method for directly detecting the responses of specific cell types by incorporating living cells into sensor technology. A notable example is the work of [58], who developed a magnetic cell-based sensing platform. This system integrates an LC cross-coupled inductive sensor with differentiated cardiac progenitor cells on a chip. The innovative design allows for the real-time observation of the pulsatile movements of these cells, paving the way for enhanced monitoring and analysis of cellular behavior.

The demand for at-home and PoC medical diagnostic tools is increasing, driven by their ability to deliver quick and affordable personal healthcare solutions. To meet this requirement, an enhanced version of the 1 GHz LC cross-coupled inductive sensor is introduced [59]. This new design features a handheld reusable reader and a disposable open-well cartridge that are specifically tailored for the detection of nucleic acids and antigens in point-of-care (PoC) applications. This diagnostic system incorporates an innovative “magnetic freezing” technique. This approach significantly reduces measurement time, removes the need for baseline measurements before or during biological assays, and effectively minimizes sensor noise.

The reviewed LC inductive magnetic sensors, while offering benefits in terms of cost and sensitivity, face limitations in their ability to distinguish between different types of magnetic particles. In [60], the authors addressed this challenge through their innovative CMOS magnetic spectroscopy relying on the magnetic susceptibility concept that works in a range of 1.1 GHz to 3.3 GHz. Magnetic susceptibility [61] is frequency-dependent and varies by particle size, leading to a unique magnetic frequency spectrum, or “signature,” that allows for distinguishing between different bead types used for analyte detection.

Notably, to achieve robust performance and reliable design across variations in process, voltage, and temperature, the LC cross-coupled oscillator can be implemented in feedback structures such as a phase-locked loop (PLL) or designed with a compensation circuit [62]. Alternatively, a current-reuse LC architecture that has ultra-low power operation [63], combined with adaptive control techniques [64], can serve as the core structure for an inductive sensor, resulting in enhanced performance while reducing power consumption.

Biomolecules play a critical role in regulating biochemical and biological processes, including genetic coding and cellular function. The increasing demand for advanced instrumentation capable of managing extensive libraries of biomolecules under controlled conditions highlights significant limitations in existing platforms. These limitations are particularly evident in areas such as multiplexing capabilities, microenvironment regulation, and prevention of cross-contamination. In response to these challenges, a fully integrated ferrofluidic multifunctional platform based on CMOS technology, specifically designed for biomolecular processing, is presented by the authors of [22]. This cutting-edge platform integrates a variety of functionalities, featuring a range of in-pixel electrochemical sensors and actuators, coil-based magnetic manipulators, temperature regulators, and sensors, as well as magnetic sensors, all on a single CMOS chip fabricated in a 45 nm PDSOI CMOS technology. The functionality of the biochip is outlined as follows. Ferrofluidic droplets are injected from micro dispensers, with each droplet containing magnetic particles suspended in a standard buffer solution (1× DPBS). These droplets are isolated using fluorinated oil (Pico-Surf1, Sphere Fluidics) to enable multiplexing while minimizing cross-contamination. The on-chip ferrofluidic multifunctional pixels generate a collective magnetic field that facilitates the manipulation of droplets both within the pixels and between adjacent pixels. The concentration of magnetic beads in the droplets is monitored using LC inductive cross-coupled magnetic sensors to ensure their manipulability by the on-chip ferrofluidic pixels. Subsequently, the droplets are moved to the targeted ferrofluidic pixels via the generated magnetic field. In these pixels, integrated electrochemical sensors and temperature regulators analyze the molecular analytes present in the droplets. Finally, the processed ferrofluidic droplets are transported to the outlet. The sensing inductor of the magnetic sensor is designed in a bowl shape to ensure uniform sensing, regardless of the location of the magnetic beads [56]. The magnetic sensor exhibits a measured sensitivity of 57.3 kHz/(beads/nL) for concentrations up to 200 beads/nL, beyond which the sensitivity begins to decline. This work demonstrates the platform’s capabilities through the use of recombinase polymerase amplification (RPA) of DNA, highlighting real-time monitoring of molecular interactions. This innovative approach emphasizes the potential of CMOS technology to revolutionize biomolecular analysis, offering a compact and efficient solution for a range of applications in molecular diagnostics and biochemistry.

For cell-sorting applications, integrated transformers are particularly well suited. However, they necessitate the use of additional tuning inductors to attain the desired sensitivity for individual beads [65]. In [66], to manage this challenge, the authors developed a coupled inductive bridge for magnetic sensing applications. The bridge sensor, implemented using a 65 nm bulk CMOS process, operates in the frequency range of 770 MHz to 1450 MHz. It effectively provides consistent and reliable detection of iron-oxide magnetic beads measuring 4.5 μm.

#### 2.2.2. Hall Effect Sensors

**Figure 4:**
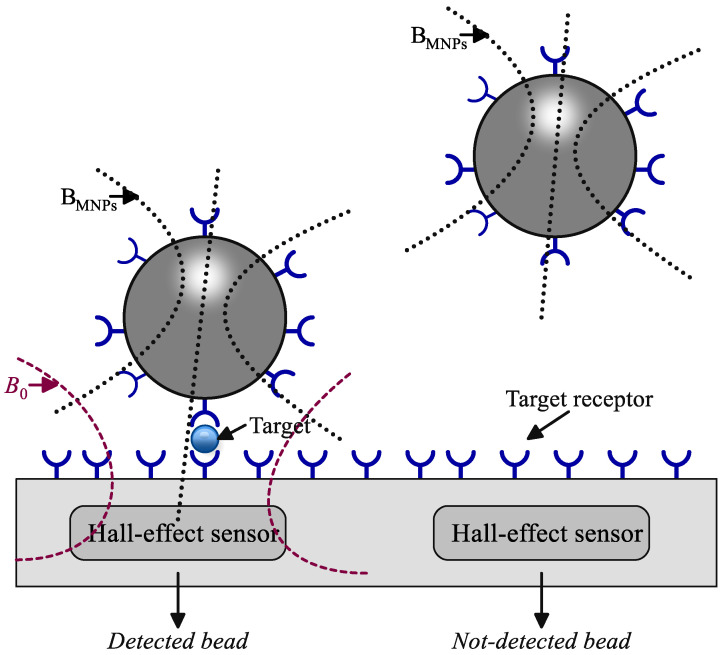
Cross section of a Hall effect sensor for magnetic bead detection [49].

The Hall effect, identified by Edwin Hall in 1879 [67], characterizes the behavior of electric charges when subjected to both electric and magnetic fields. The external magnetic field deflected charge carriers in a conductor from their usual current path. This creates a transverse electric field that halts the carrier drift, resulting in a measurable transverse electric potential known as Hall voltage. This phenomenon is referred to as the Hall effect and serves as a fundamental principle in the design of Hall effect sensors for detecting magnetic particles. In Hall effect sensors, as illustrated in Figure 4, magnetic beads (consisting of MNPs) are initially anchored to the sensor surface using specially designed affinity-based assays. An external magnetic field (*B*_0_) is subsequently applied to magnetize the bead, generating a stray magnetic field. The Hall sensor then measures the interplay between the magnetic fields, allowing for the identification of the particles.

**Figure 5:**
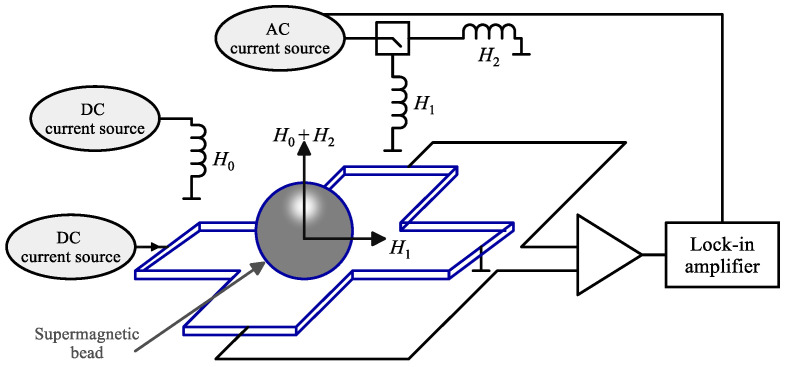
Schematic overview of the measurement setup for detecting a single magnetic microbead with a silicon Hall effect sensor.

Magnetic Hall sensors can be easily integrated and scaled within CMOS technology, allowing for the development of compact and cost-effective systems. The advancement of Hall sensors for detecting superparamagnetic beads in biomedical applications was initiated by the authors of [68]. It was the first to successfully detect a single 2.8 μm Dynabead using a Hall sensor fabricated in 0.8 µm CMOS technology. In their study, the authors employed a phase-sensitive detection method in which a direct current (DC) magnetic field (*H*_0_) was applied perpendicularly to the sensor plane to magnetize the bead, while an alternating current (AC) field induced oscillations in the bead’s magnetic moment introduced either in the x-direction (*H*_1_) or z-direction (*H*_2_) as shown Figure 5. The Hall voltage (*V*_H_), which contains a component proportional to the magnetic induction produced by the bead, is measured with a lock-in amplifier. By incrementally adjusting the DC field, they observed corresponding changes in the magnitude of the AC Hall voltage, which served as evidence for the presence of the bead.

**Figure 6:**
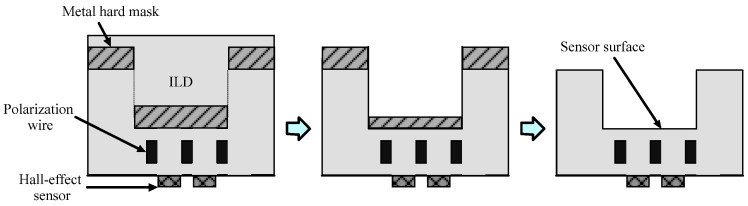
Post-processing etching steps conducted after CMOS fabrication aim to minimize the distance between the magnetic sensing region and the Hall sensors.

To obtain measurable signals from Hall effect sensors, it is essential to minimize the distance between the magnetic particles and the sensor surface [69]. A design introduced by the authors of [70] focused on minimizing the distance between beads and sensors by employing reactive ion etching on the inter-layer dielectric (ILD) above the sensor area as depicted in Figure 6. Additionally, an optional metal wet etching process was utilized to remove the metal hard mask, bringing the beads an extra 0.5 μm closer to the Hall sensor array. The design includes a 64-unit Hall effect sensor array fabricated using a 0.18 μm CMOS process, enabling it to detect 4.5 μm beads (Dynabeads^®^ M450) across its entire surface. In another study [71], the ILD above the sensor area was etched, effectively reducing the distance from 9 µm to 3 µm. This modification resulted in a remarkable enhancement in signal strength, increasing it by a factor of 10 to 20 for magnetic beads approximately 1 µm in size.

**Figure 7:**
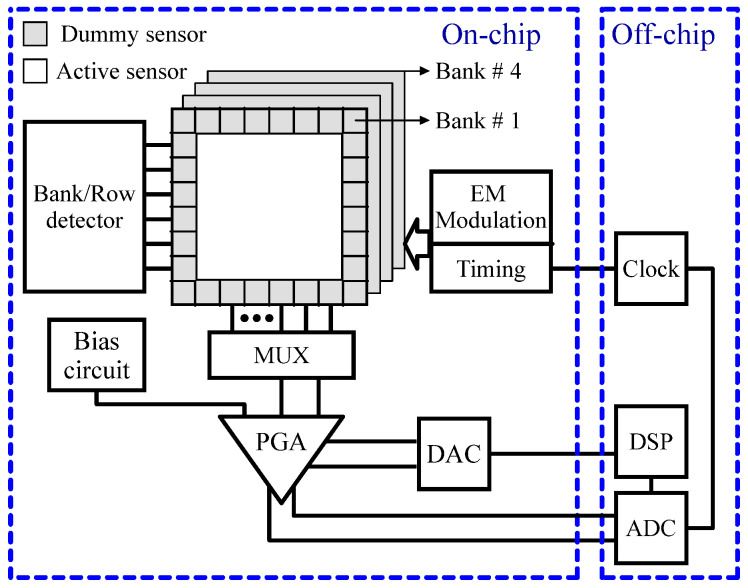
The block diagram of the array of Hall effect magnetic sensors based on magnetic relaxation [49].

To resolve the magnetic field generated by a magnetic bead in the presence of a significantly larger magnetizing field baseline, it is essential to utilize reference sensors, perform baseline calibration, and ensure temperature stabilization. However, these requirements can lead to considerable drawbacks in terms of on-chip area, power consumption, and detection time. A promising alternative to mitigate these challenges is the use of relaxation measurements, which capture the intrinsic magnetization dynamics of the beads and enable measurements that ideally do not depend on a baseline [49]. In this technique, the bead is first magnetized using a strong magnetic field. The magnetizing field is then rapidly removed, and detection begins, effectively eliminating the substantial baseline and allowing for the measurement of the decaying magnetic field emitted by the bead. The authors of [49] proposed a CMOS Hall effect magnetic label detector tailored for biomedical assays, which utilizes the magnetic relaxation signature from microbead labels for detection. This innovative method enhances tolerance to environmental fluctuations and alleviates dynamic range requirements, thereby removing the necessity for baseline calibration and reference sensors. Figure 7 illustrates the block diagram of the prototype magnetic bead detector chip fabricated using 0.18 µm CMOS technology. Given that micron-sized beads produce only localized magnetic fields, a single large-area sensor would be ineffective. Consequently, the magnetic sensor array is structured into four banks, each consisting of 64 individual Hall effect devices. Within each bank, active sensors are strategically positioned alongside dummy sensors to reduce variations between sensors and mitigate thermal non-uniformity. In this prototype, sensor outputs are processed serially through a multiplexed readout channel, which performs critical functions such as offset rejection and signal amplification. The incorporation of embedded electromagnets within the device eliminates the need for external magnetic sources, thereby decreasing power dissipation. To ensure high accuracy, correlated double sampling (CDS), along with offset servo loops and magnetic field modulation techniques, effectively minimizes the detector offset to sub-µT levels. As a result, the system can successfully detect single 4.5 µm magnetic beads within 16 ms, maintaining a probability of error below 0.1%.

In another attempt, the authors of [72] engineered a CMOS Hall effect sensor specifically for characterizing and detecting MNPs using a magnetic relaxation technique. This innovative sensor features embedded wires that create a localized magnetizing field, which is quickly deactivated to monitor the relaxation of the MNPs. The integrated high-speed readout electronics within the CMOS chip allow for seamless integration with microfluidic systems, making it ideal for lab-on-a-chip (LoC) setups and PoC applications.

In the realm of magnetic immunoassays, detecting and counting multiple magnetic particles simultaneously is crucial. Significant research efforts have focused on integrating dense arrays of Hall cross sensors, each capable of detecting a single magnetic particle specifically bound to its surface [17,70,73]. A notable example of this work is presented by the authors of [17], who developed a CMOS Hall sensor featuring 10240 sensing sites. They validated the sensor’s functionality using Dynabeads ranging from 1 μm to 4.5 μm, detected through a magnetic relaxation technique. For practical applications, the sensor array was coated with a known concentration of Human Serum Albumin (HSA). Magnetic beads were functionalized for specific binding. Then, they were incubated on the array surface and subsequently counted. This design reduced the readout time by a factor of four compared to that of their previous work [49] by implementing a parallel readout interface instead of a single readout. Additionally, the sensitivity of the magnetic beads was improved by more than 50 times compared to a single Hall sensor.

Hall sensors combined with microfluidics have proven effective in various biological applications [74]. In [75], the authors developed a Hall sensor using a 0.35 µm CMOS process specifically for testing human whole blood. A human blood sample containing the target biomolecules is introduced onto the proposed system-on-chip (SoC). The testing process involves several automated steps, including blood filtration, biomolecular conjugation, electrolytic pumping, magnetic flushing, and detection, all managed by a microcontroller unit (MCU). The biomolecular signal is then converted into an electrical signal by a CMOS-based Hall sensor array, which is coated with a biomolecular probe. To enhance usability, the system integrates four light-emitting diodes (LEDs) and a battery to indicate the detection steps, facilitating a user-friendly self-test PoC application.

Human biomagnetic fields, emitted by our bodies, can indicate health and physiological changes [76,77,78]. Long-term monitoring often requires participants to wear sensors in their daily lives. To minimize disruption, these sensors should be small, lightweight, and affordable, utilizing SoC technology for better integration. Low-cost biomagnetic wearable sensors effectively fulfill these requirements. In a different approach, the authors of [79] developed a low-cost, highly reliable, wearable biomagnetic sensor based on a CMOS Hall effect sensor, featuring synchronous excitation for sensitivity calibration. This approach effectively mitigates the inherent non-ideal drift typical of Hall probes. By employing an on-chip coil, synchronous excitation generates a DC reference magnetic voltage at the Hall probe’s output, achieved by modulating the coil’s frequency to match the Hall spin-current bias frequency. Experimental results demonstrate a sensitivity drift of just 73 ppm per degree Celsius. Another study proposed a wearable CMOS Hall effect magnetic sensor to record small magnetic fields produced by the electrical activity of muscles [80].

#### 2.2.3. GMR Sensors

**Figure 8:**
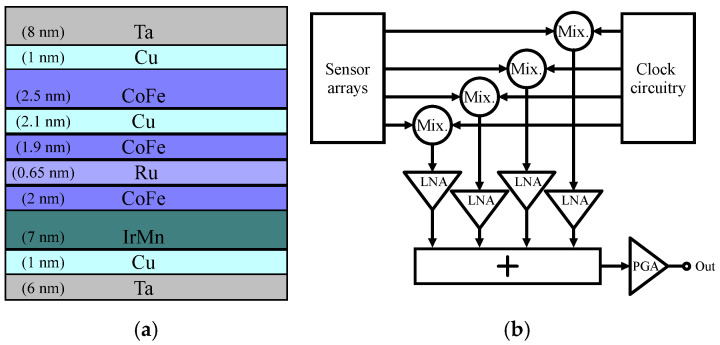
(**a**) Illustrative diagram of the GMR SV sensor film stack and (**b**) the readout circuit.

The GMR effect relies on the differing scattering processes of spin-up and spin-down conduction electrons as they traverse multilayer structures composed of ferromagnetic and nonmagnetic materials [81]. In these structures, the alignment of the ferromagnetic layers, either parallel or antiparallel, can be carefully manipulated. By applying a magnetic field, the relative orientation of the magnetizations in the two ferromagnetic layers can be altered. When the magnetizations align, the electrical resistance of the system decreases. Conversely, when the magnetizations are aligned antiparallel, the resistance increases. This phenomenon underpins the functionality of GMR sensors, which can identify magnetic particles and serve as a foundation for creating biochips [82]. A commonly utilized configuration of GMR sensors is the spin-valve (SV) sensor, which is known for its high sensitivity at room temperature and its straightforward fabrication process with CMOS technology [21,83,84,85,86,87,88]. The SV usually consists of a pinned layer that has a stable magnetization, alongside a free layer that allows its magnetization to adjust freely in response to an external magnetic field during operation. For instance, the authors of [83] developed a 1008 array (16 subarrays) of GMR-SV sensors that was implemented in a 0.25 μm BiCMOS process to detect DNA hybridization. Figure 8a illustrates the top view of the sensor, along with the annotated thicknesses of the GMR-SV sensor film stack integrated with a CMOS chip. When MNPs bind to the hybridized DNA, the resistance of the sensor changes. The resulting electrical signals are then measured directly using the readout circuit that is illustrated in Figure 8b. In this study, frequency-division multiplexing (FDM) and time-division multiplexing (TDM) techniques are employed to reduce the signal readout time from each array. A single 42 kHz master clock generates four carrier frequencies through the clock circuitry for the mixers. This modulation enables FDM, to shift the signal spectrum upward to minimize the impact of 1/*f* noise in subsequent circuits and effectively separate the desired signals from electromagnetic interference (EMI). The low-noise amplifier (LNA) offers a gain of 18, with an input-referred noise level below 50 nV/√Hz. A programmable-gain amplifier (PGA) with selectable gains of 1, 10, or 100 is incorporated to optimize the system’s dynamic range. The final output is digitized by an off-chip analog-to-digital converter (ADC), and tone measurements are conducted using a fast Fourier transform (FFT) analysis [89]. The GMR-SV sensor is capable of identifying approximately 100 magnetic nanotags for each sensor during DNA hybridization assays conducted at a DNA concentration of 10 nM.

Another GMR-SV biosensor was fabricated by the authors of [85] using a 0.18 μm CMOS process, demonstrating protein detection capabilities at a concentration of 6 pM. This design offers several advantages over previously reviewed work. For instance, it employs voltage excitation instead of current excitation, which facilitates the multiplexing of sensors by summing their currents without requiring a dedicated amplifier for each sensor. Also, the sensor count has increased from 16 to 256. Furthermore, this design achieves a lower readout time thanks to the combination of FDM and TDM with parallel readout channels. In the examined work [85], MNP detection is conducted using standalone GMR sensor chips, with the associated electronics located on separate chips. This arrangement restricts the number of GMR sensors and reduces the system’s tolerance EMI. To address this limitation, the authors of [90] integrate 192 GMR-SV sensors onto a CMOS interface, achieving high yield, low power consumption, and high sensitivity for biomolecular recognition applications using AMS 0.35 μm technology. The design’s low circuit noise enables a maximum equivalent magnetic noise of 11.5 nT/√Hz, allowing the full system to detect 250 nm magnetic nanoparticles with only a −1.4 dB degradation in the circuit-imposed signal-to-noise ratio (SNR).

In the reviewed studies [83,85,90], the detection of MNPs tethered to the sensor surface has relied on local perturbations in the magnetic field. This approach, while sensitive, demands high uniformity in the magnetic field, intricate modulation schemes to manage the low signal-to-baseline ratio, and prolonged measurement times to mitigate noise [86]. Overcoming the mentioned issue relaxation concept (the same concept is explained in the section of the Hall effect sensor) is accomplished in the GMR sensor. In magnetorelaxometry (MRX), MNPs are initially magnetized, and their temporal response is subsequently monitored after the magnetic field is removed. This sensing technique is not affected by variations in magnetic field homogeneity, making it well suited for low-power portable applications. In [91], the authors developed an 8 × 10 CMOS GMR-SV sensor based on the relaxation concept that was the first GMR sensor to have been reported for an MRX bioassay. This technique enables the sensor to effectively monitor the rapid relaxation processes of 30 nm MNPs. Additionally, this design employs a magnetic correlated double sampling (MCDS) technique to eliminate variations between sensors, compensate for temperature drift, address circuit offsets, and mitigate non-linearity. In this study, an off-chip power amplifier (PA) and a Helmholtz coil are employed to generate a pulsed magnetic field for the sensor chip. The PA generates a DC current, which is converted into a pulsed current for the Helmholtz coil using an electromagnet driver. Subsequently, the analog front end (AFE) captures the sensor’s response and transmits the data to a PC for post-processing. More details about the electronic design of the sensor are prepared in [86].

Although the MRX method removes the baseline-to-signal ratio and is resistant to sensor mismatch when employing MCDS [86], the relaxation timescale of MNPs varies significantly, ranging from nanoseconds to seconds depending on their size [91,92]. On the other hand, the detection of slow relaxation signals involves considerable 1/*f* noise [86] while the detection of fast relaxation signals necessitates the use of a high-speed AFE along with rapid, pulsed magnetic fields [93]. Therefore, the design of the AFE is a challenging issue to satisfy readout time, dynamic range, area, and power consumption. To address these challenges, the authors of [93] introduced an AFE designed using a 0.18 μm CMOS process. This AFE incorporates innovative architectural and circuit-level techniques, such as fast settling duty-cycle resistors to decrease readout time and a high-frequency interference rejection sampling technique integrated into the ADC to alleviate the dynamic range requirement. The AFE demonstrates an input-referred noise of 46.4 nT/√Hz, an input-referred baseline of less than 0.235 mT, and a readout time of 11 ms, all while consuming only 1.39 mW. In another work, the same researchers designed a low-noise magnetic sensor front end featuring an 18-bit Zoom ADC designed for the detection of temporal MNP relaxation [21].

It is worth mentioning that although the study of [86] is effective for measuring signals from sensor arrays, the use of an external Helmholtz coil and PA entails significant power consumption and a large form factor. In [94], the authors tried to provide more integration by utilizing on-chip pulsed excitation and a readout circuit that employed the MCDS technique for a scalable GMR biosensor array. The system features a 12 array of GMR-SV sensors integrated with field-generating strip-line inductors located directly beneath the sensors on the same chip, along with a specialized AFE. By incorporating the strip lines on-chip, this design removes the necessity for an external, bulky coil and its associated PA, significantly enhancing scalability. The on-chip field generators are responsible for magnetizing MNPs that are anchored to the surface through a sandwich immunoassay, consisting of a capture antibody, the target analyte, and a detection antibody linked to an MNP. The MNPs that are captured create a localized magnetic field, which is detected by the underlying GMR sensor. Custom electronics employing the MCDS technique are utilized to minimize flicker (1/*f*) noise and offset, allowing for the extraction of subtle signals of interest in the time domain and shortening the readout time while maintaining a high sensitivity level. Measurements indicate the capability to detect MNPs at an impressively low signal level of 6.92 ppm.

#### 2.2.4. NMR Sensors

NMR involves the interaction of radio frequency (RF) magnetic fields (*B*_1_) with atomic nuclei, such as hydrogen protons in the presence of a static magnetic field (*B*_0_). So, an NMR system needs to include a magnet to generate a static magnetic field, *B*_0_, and an RF transceiver connected to the RF coil in which the coil surrounds a sample to generate an RF magnetic field, *B*_1,_ and to monitor the resonance. When target biological entities are present in an aqueous solution, they influence the behavior of hydrogen protons, allowing for their detection through NMR experiments. This bio-sensing technique based on NMR was introduced by the authors of [95].

**Figure 9:**
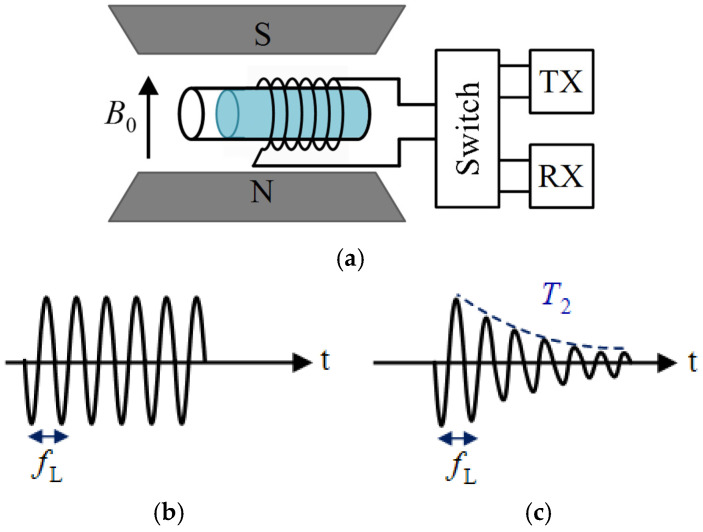
The fundamental principle operation of the NMR system. (**a**) The core of the NMR system; (**b**) the switch is connected to the TX, and B_1_ is generated to excite the sample; (**c**) the switch is connected to the RX, and the spins are relaxed by the relaxation time of T_2_.

The operational principle of the NMR system is as follows (see Figure 9). In the first step, an RF signal at a specific frequency of *ω*_L_ (Larmor frequency) is transmitted to the coil, generating an RF magnetic field, *B*_1_, that resonantly excites the nuclear spins within the sample. After the RF excitation is turned off by switching the coil to receiver (RX) mode, the spins precess around the *B*_0_ axis at the frequency *ω*_L_ (where *ω*_L_ = *γB*_0_, with *γ* denoting the gyromagnetic ratio). During this precession, the spins gradually lose phase coherence due to spin–spin interactions, resulting in exponential relaxation of the net magnetic moment. As the spins precess and relax, the coil detects an NMR signal, which appears as a damped sinusoidal waveform characterized by the spin–spin relaxation time, *T*_2_. NMR systems are typically designed as relaxometry, spectrometry, or magnetic resonance imaging (MRI) devices. In relaxometry, the primary goal is to measure *T*_2_, as the rate of phase decoherence varies among materials containing ^1^H protons. Spectrometers focus on measuring and identifying the spectrum and its components, while MRI utilizes *T*_2_ measurements at various locations within a body part to create a spatial heat map of *T*_2_.

**Figure 10:**
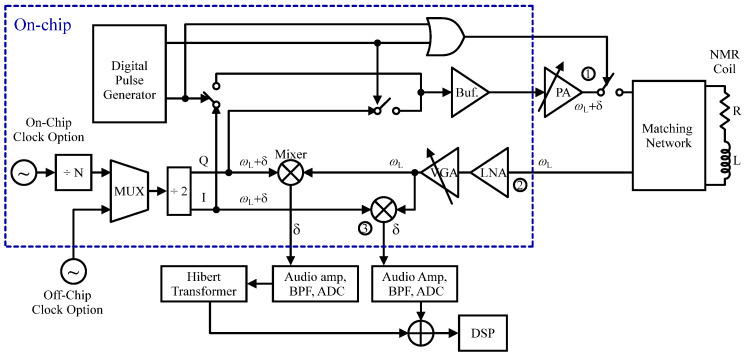
The structure of the NMR system consisted of an RF transceiver, a portable permanent magnet, and in-house fabricated 500 nH planar microcoils.

NMR-based detection has emerged as a highly effective analytical tool across various fields especially in biosensing. For instance, the authors of [96] developed an NMR relaxometry biosensor for the detection and molecular analysis of cells that utilized an on-board RF transceiver making the system bulky. To reduce the overall footprint in a subsequent effort, the authors of [97] integrated the RF transceiver designed at the Larmor frequency of 21.3 MHz using a 0.18 µm CMOS process. This integration led to a more compact NMR relaxometry system capable of detecting biological targets. Figure 10 illustrates the architecture of the whole NMR system which consisted of an RF transceiver, a portable permanent magnet to generate *B*_0_ in the range of 0.1–0.5 T, and in-house fabricated 500 nH planar microcoils. In the RX part, a weak spin-echo signal is picked up at node 2, amplified by a low LNA and a variable gain amplifier (VGA), and then down-converted using mixers and quadrature local oscillator signals. In the TX part, the excitation RF magnetic field is created by the same local oscillators, with an off-chip PA. Field inhomogeneity leads to rapid damping of the NMR signal, complicating true *T*_2_ measurement despite high RX sensitivity. To address this, a digital pulse generator in the TX transmits an RF signal sequence that offsets field inhomogeneity, yielding accurate *T*_2_ measurements. The absence of avidin shows a *T*_2_ value of 73 ms, while its presence reduces *T*_2_ to 31 ms, indicating successful avidin detection. To provide more integration, in another design by the authors of [12], the coil is designed as an on-chip, which leads to significant size reduction in the NMR sensor and makes it more compatible with LoC applications. It is worth noticing that large magnets produce stronger NMR signals that relax sensitivity requirements for transceiver design but make bulkier systems. So to achieve a compact NMR system with a small magnet size, a high-performance CMOS RF transceiver is required that is considered in [12].

Implementing a CMOS NMR system with a portable magnet in addition to size faces significant challenges of the variations in the *B*_0_ field. This necessitates a robust calibration scheme, as conventional frequency stabilization techniques may not be effective amid large *B*_0_ fluctuations. To resolve this concern, in [98], the authors proposed a *B*_0_-field stabilization scheme for a handheld µNMR CMOS platform using a portable 0.46 T magnet. The authors integrated a Hall sensor to detect variations in the *B*_0_ field of the permanent magnet, which must be oriented orthogonally to the RF magnetic field. The detected *B*_0_-field variations modulate the current through the magnet’s auxiliary coil, stabilizing the resultant magnetic field and enabling system-level calibration. Also, this design provides outstanding performance such as utilizing a single CMOS chip to perform versatile and sensitive chemical and biological assays on diverse unprocessed samples, including proteins, DNA, and polymers.

One of the key requirements in the design of bio-sensing systems is the ability to sense multiple samples simultaneously, which facilitates higher throughput and enables real-time comparison of results. To deal with this issue, many researchers suggest utilizing digital microfluidics (DMFs) [99,100,101]. In one attempt, the authors of [101] developed an NMR system with a DMF device integrated inside a portable magnet that enables efficient multi-sample handling. In fact, with the use of DMFs, multiple samples can time-share a single NMR sensing site aligned with the transceiver’s coil, thereby enhancing throughput and repeatability while minimizing the risk of assay contamination.

**Figure 11:**
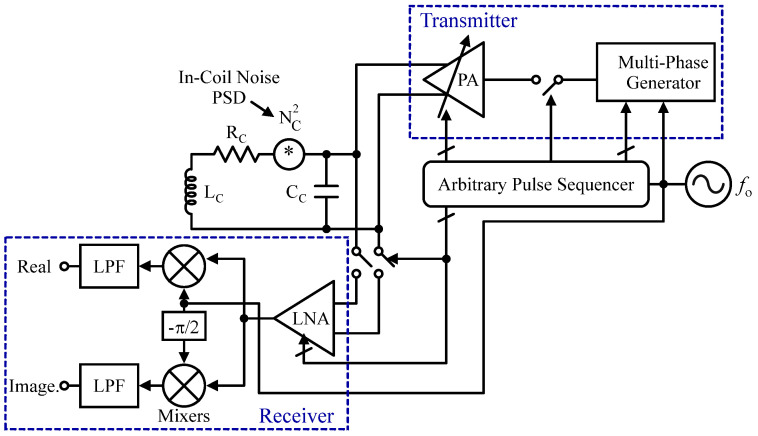
The architecture of spectrometer.

As mentioned previously, an NMR system can be implemented for spectroscopy. For example, the authors of [102] developed a 5–300 MHz CMOS transceiver in 130 nm CMOS for multi-nuclear NMR spectroscopy dedicated to drug discovery which spectroscopy limited to 1D [102]. In a different effort, in [103], the authors reported the first µNMR system capable of 1D and 2D spectroscopy using 0.18 µm CMOS technology. It features a 0.51 T magnet with a six-direction electrical shim and 0.13 ppm homogeneity, paired with a 1 mm external NMR coil for improved sensitivity. As depicted in Figure 11, the design includes an RF transceiver and an arbitrary pulse sequencer. The RX amplifies signals from nuclear spin precessions, while the TX excites spins using a multiphase generator and PA, allowing for versatile applications in NMR spectroscopy and relaxometry. For parallel spectroscopy, in [104], a fully integrated multi-channel system at 300 MHz is introduced. In a separate effort [105], a 21 MHz differential NMR spectrometer using a 0.13 µm CMOS process is developed. The system features a dual-path RX, two off-chip solenoid mini-coils, and off-chip pulse generators to excite the RF coils. The authors of [106] presented a broadband single-chip CMOS transceiver compatible with microcoils and external resonators for multi-nuclear spectroscopy. In one other attempt, a compact wideband CMOS NMR spectrometer capable of multinuclear molecular fingerprinting is introduced [23].

Recently, MRI has garnered attention alongside NMR spectroscopy and relaxometry. For example, the authors of [107] developed a portable high-resolution multidimensional NMR system that is capable of all three key NMR modalities (relaxometry, spectroscopy, and MRI). It can measure 18 samples simultaneously using a shared coil and a single silicon chip. In another study [108], the authors presented the first fully integrated MRI RX designed for on-coil operation, featuring a quadrature down-conversion RX for simultaneous signal digitization from two coils. Its functionality was validated through imaging of a human wrist. In [20], the researchers introduced an implantable needle-shaped NMR sensor using 0.13 µm CMOS technology, enabling in vivo studies of brain physiology with a 100-fold improvement in temporal resolution for blood-oxygenation-level-dependent (BOLD) effect detection. This sensor features a quadrature RX, PLL synthesizer, and a TX path with an H-bridge PA, consuming 20 mW and providing precise volume sensitivity with magnetic resonance images at 13 µm isotropic resolution.

## 3. CMOS-Based Optical Sensors

With the rapid advancements in biotechnology and the increasing need for accurate and real-time diagnostic tools, CMOS technology has emerged as a pivotal foundation for developing innovative optical biosensors. These biosensors leverage the sensitivity, reliability, and specificity of optical detection methods, combined with the scalability and integration capabilities of CMOS technology, to create highly efficient and compact devices for various biomedical applications. The optical sensor detects biological interactions by measuring changes in light properties including intensity, wavelength, or refractive index okcorrelating with analyte concentration.

The literature documents various optical biosensor structures, including fluorescence, bioluminescence, absorption photometry, silicon photonics, surface-enhanced Raman scattering, and plasmon-based biosensors. The most prevalent optical detection methods that can be implemented with CMOS technology include fluorescence, bioluminescence, and evanescent wave techniques. Each method generates optical signals differently based on the analytes through a label-based or label-free detection process that will be briefly discussed in the upcoming sections.

### 3.1. Sensing Principle of Optical Sensors

**Figure 12:**
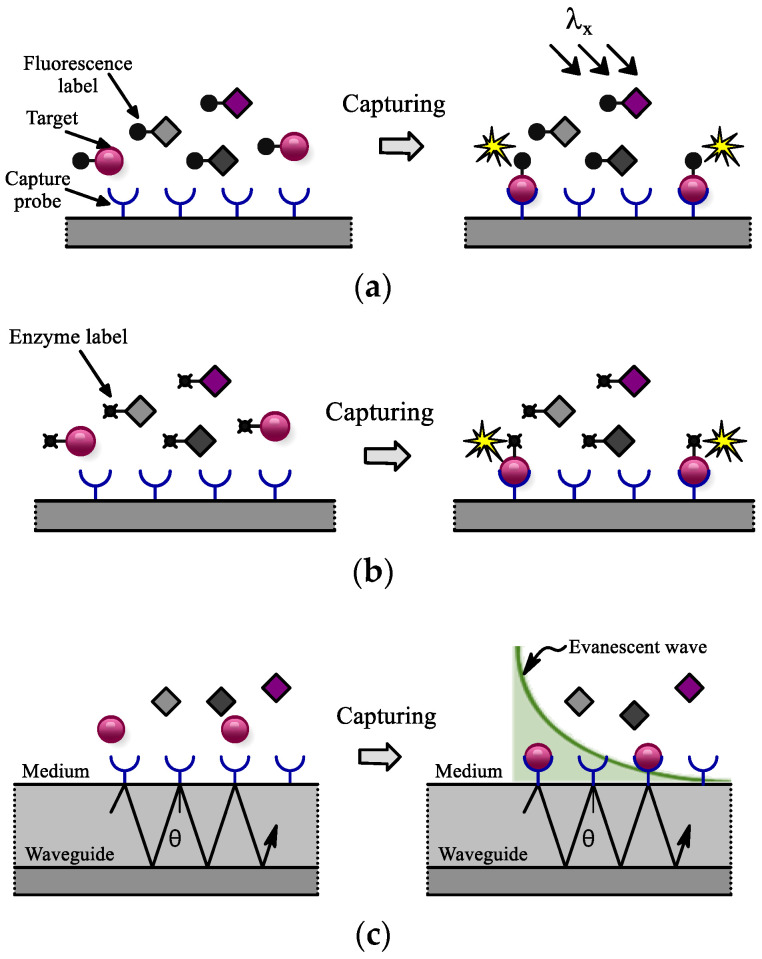
Optical detection principle. (**a**) Fluorescence, (**b**) bioluminescence, (**c**) evanescence wave.

In fluorescence-based biosensors, as depicted in Figure 12a, fluorophores as label targets absorb excitation light at a specific wavelength and, after binding to immobilized probes, emit photons at a different wavelength. This interaction produces an optical signal that can be detected by photosensitive elements after filtering out background light. Bioluminescence biosensors (Figure 12b) rely on a chemical reaction for signal photoemission, eliminating the need for an excitation source or filter, which reduces background noise. This makes bioluminescence a cost-effective and easily integrated solution for LoC applications compared to fluorescence methods. Evanescent field detection is a popular label-free method for optical biosensors, enabling real-time monitoring of molecular interactions (Figure 12c). It involves a receptor layer on a waveguide surface where the waveguide’s refractive index is higher than that of the surrounding medium. When light strikes the waveguide at an angle exceeding the critical angle, an evanescent wave penetrates slightly into the lower index medium, allowing for interaction detection between the receptor and analyte. This approach limits interaction with materials adjacent to the surface, avoiding interference from unbound substances in the solution. Silicon photonic biosensors are commonly used in this method and are compatible with CMOS technology.

### 3.2. CMOS-Based Interface Circuit of Optical Sensors

#### 3.2.1. Fluorescence Sensors

Fluorescence sensing has emerged as a pivotal technology in biomolecular detection, providing sensitive, specific, and robust capabilities in diagnostic and medical research. However, this approach typically relies on external components, such as an excitation source for the photoemission of fluorophores and filters to effectively isolate the emitted signals from background interference with a high rejection ratio. By incorporating the essential elements of fluorescence sensing into CMOS technology, several advantages can be achieved, including reduced costs, high integration density, and enhanced signal processing flexibility. In [109], the authors developed a fluorescent-based biosensor for DNA microarrays that comprised a transducer array, molecular capture probes, and advanced readout circuitry. This system enables real-time detection of fluorescent signals from biological targets, allowing for the study of DNA hybridization kinetics across varying concentrations. In another attempt to provide more integration, a copper-based nanoplasmonic filter in a 65 nm CMOS process is presented [110]. It enables a fully integrated fluorescence biosensor capable of conducting multiplexed assays with high sensitivity, detecting 48 zeptomoles of Qdot-based fluorophores.

**Figure 13:**
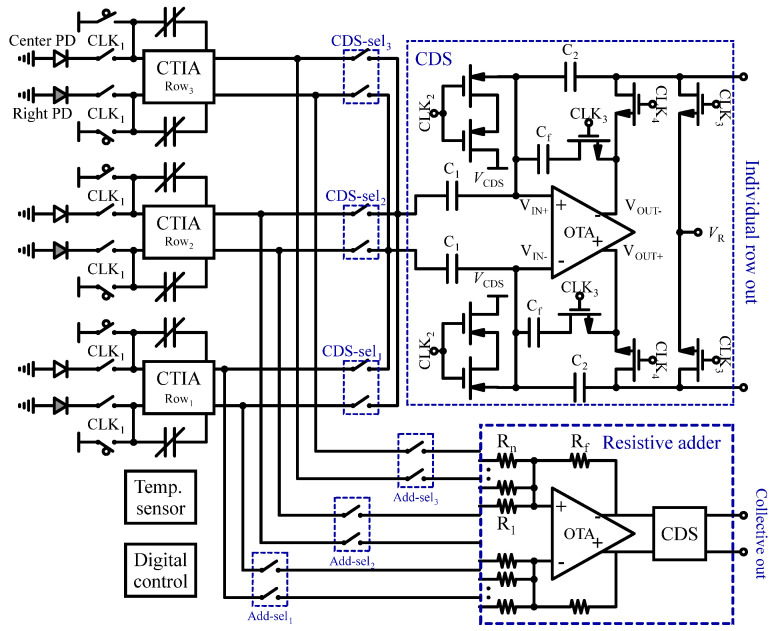
Readout circuit of a fully integrated fluorescence-based sensor for dynamic monitoring of living cells.

In another study [111], a fully integrated fluorescence sensor designed for the dynamic monitoring of living cells is introduced. This innovative design, detailed in Figure 13, features on-chip bandpass optical filters, photodiodes, a capacitive transimpedance amplifier (CTIA), CDS, and a resistive adder block, all implemented in 65 nm standard CMOS technology. The study effectively captures signals from various optical filters and photodiodes arranged differentially across the central and left/right columns of the chip. This is accomplished by utilizing six rows of front-end circuitry. Each row employs a CTIA with a variable feedback capacitor to convert the differential photodiode current into a voltage signal. To mitigate low-frequency correlated noise and offsets, the CDS circuit samples the CTIA output twice per row. To optimize area and minimize power consumption, a single CDS block is shared among every four rows through the use of CDS-Sel switches. Additionally, a resistive adder is integrated to combine the outputs from multiple rows, enhancing overall signal strength. The sensor also includes an on-chip thermal sensor, along with a clock generator and a digital scan chain block, facilitating efficient distribution of all digital control signals throughout the circuit.

In a study by the authors of [112], an advanced optoelectronic biosensor was developed for wired implantable fiber photometry applications using a 0.18 μm CMOS process. This research integrated a differential CMOS photodetector with a differential CTIA (DCTIA) to create an input photo-sensing module. Additionally, a second order 1-bit continuous-time delta-sigma modulator (CTΔΣM) was employed to achieve a wide dynamic range, streamlined implementation, and high energy efficiency. Similarly, in [113] another wired implantable sensor for optical communication with brain cells is introduced. While the reviewed wired implantable sensors offer high performance, their effectiveness for continuous monitoring is limited by the infection risk associated with external wiring. The first wireless implantable fluorescence image sensor is developed by the authors of [114], comprising a power management unit (PMU), an imaging front end, a laser driver, and a finite-state machine (FSM) for timing management. It captures images while providing substantial data transmission capabilities. In a different study, a 15-pixel fluorescence biosensing array with integrated nanoplasmonic filters suitable for ingestible applications is introduced [115]. The design adapts to complex biological environments and effectively decodes modulated RF signals for external communication. A multicolor fluorescence image sensor for real-time cancer therapy monitoring that relies on power harvesting instead of batteries enables wireless communication and operation at significant tissue depths. In [51], the authors proposed a multicolor fluorescence-based image sensor designed to eliminate the need for battery power in cancer therapy, utilizing power harvesting for real-time monitoring. This work presents the first implantable fluorescence image sensor capable of wireless communication and power transfer, operating effectively at a depth of 5 cm. It features three-color fluorescence imaging and benefits from a highly compact form factor of just 0.09 cm^3^.

#### 3.2.2. Bioluminescence Sensors

**Figure 14:**
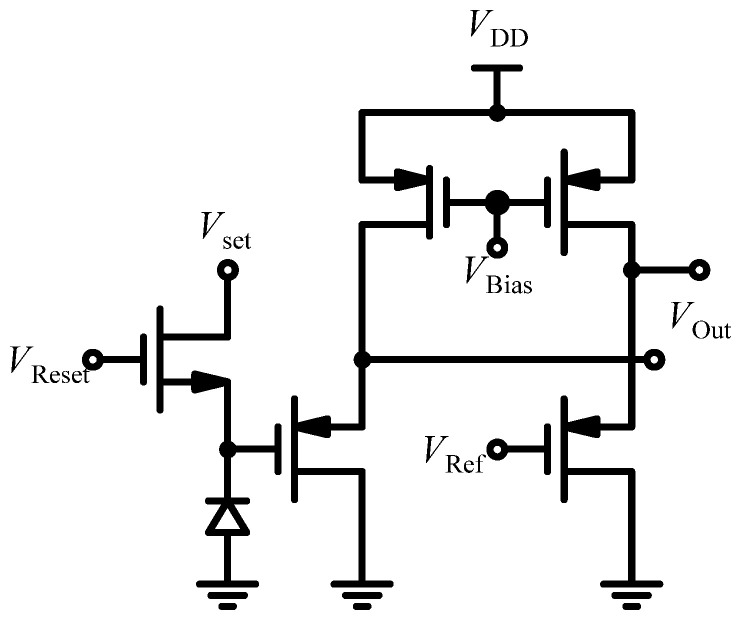
Schematic of the pseudo-differential pixel.

Bioluminescence provides several benefits compared to fluorescence assays, such as reduced background noise and the utilization of reagents with extended shelf life [116]. Additionally, it simplifies LoC integration by eliminating the need for filters and light sources [117]. For instance, in [118] the authors developed an LoC system featuring integrated bioluminescence detection for gene expression analysis, including reporter gene assays, intracellular adenosine triphosphate (ATP) measurement, and DNA sequencing. This system utilizes a straightforward fiber-optic faceplate with immobilized luminescent reporters and probes, which connects directly to an optical detection, thereby processing CMOS SoC fabricated with a 0.18 µm process. The SoC includes an 8 × 16-pixel array, a 128-channel 13-bit ADC, and a 16-column digital signal processing (DSP) single-instruction multiple-data (SIMD) array, and it consumes 26 mW. The schematic of each pixel is shown in Figure 14 and consists of a P+/N/P-sub photodiode, an NMOS reset gate, and two PMOS source followers, one connected to the photodiode and the other linked to a global reference signal, *V*_REF_. The PMOS follower circuit is employed to lower the operating voltage range of the photodiode, thereby reducing dark current and enhancing linearity. It can detect emission rates below 10^−6^ lux over a 30 s integration period at room temperature. Notably, a critical aspect of designing DNA sequencing systems is managing the low signal levels in miniaturized biological assays. Consequently, high-resolution ADC conversion is vital for accommodating the broad dynamic range of outputs from the sensor array. To tackle this, the authors of [119] developed a high-resolution ADC for miniaturized megapixel DNA sequencing arrays utilizing bioluminescence detection in 0.18 µm CMOS technology. This ADC achieves a dynamic range of 90.1 dB and a peak signal-to-noise plus distortion ratio (SNDR) of 86.3 dB at a conversion rate of 1 MSample/s, with a power consumption of 38.1 mW. This performance allows for scanning a one-megapixel sensor array in one second, which is significantly faster than the typical integration times for image sensors used to capture bioluminescence signals [120].

**Figure 15:**
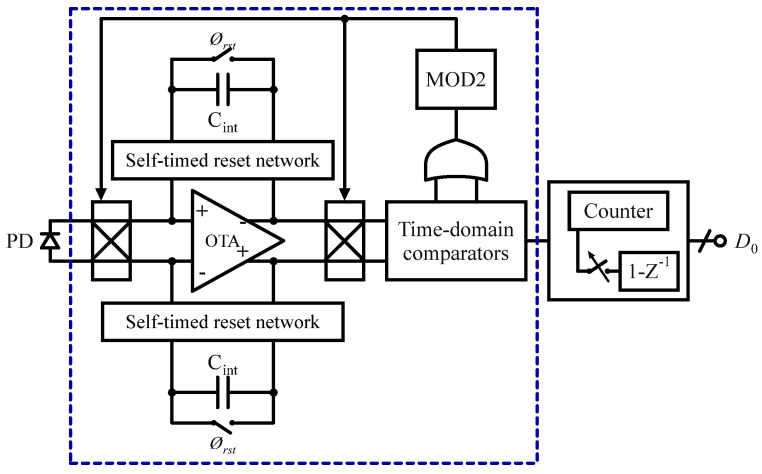
Photocurrent readout for bioluminescence detection.

In another study, a photocurrent readout with dynamic range of 124 dB for bioluminescence sensing is presented by the authors of [121] in 0.18 µm CMOS technology and is depicted in Figure 15. It features a current TIA with an asynchronous flip switch at the input, two reconfigurable time-domain all-digital comparators, and a digital counter. The outputs of the two comparators are XOR-ed to produce the necessary control signals for the asynchronous current TIA input/output flip switch. This differential readout design was primarily implemented to reduce common-mode noise, maintain balanced sensor impedance at the differential inputs, and increase the dynamic range by 6 dB compared to a single-ended configuration. Additionally, the photodiode operates in photovoltaic mode, eliminating the need for voltage biasing. This design is intended to enable in vitro NanoLuc (NLuc) luciferase-based bioluminescence sensing for the quantification of biomolecules at room temperature, with initial biological testing discussed in this article.

In a different study, the authors of [122] proposed a low-power CMOS bioluminescent bioreporter integrated circuit (IC) that was designed and fabricated in a 0.35 µm CMOS process for electronic and biological chemical sensing applications that consume only 3 mW. The core components of the IC include a microluminometer and a TX. The microluminometer features an integrated photodetector followed by an operational transconductance amplifier (OTA) and signal processor, all housed in a robust and affordable package suitable for various remote applications in hazardous environmental monitoring. The bioreporters are positioned on a CMOS IC that detects bioluminescence, processes the signals, and generates a digital output pulse with a frequency proportional to the concentration of the target substance. This digital output, which contains the sensor information, can be transmitted to a remote location either wirelessly or through a data cable. Previous studies integrated bioluminescence detectors used a CMOS-integrated photodiode followed by active pixel sensor circuits [118] and a continuous-time OTA-based integrator with high gain and low noise [122]. These fully integrated solutions necessitate a large battery, which restricts the miniaturization of the ingestible capsule. In another attempt, the authors of [123] proposed a 26 nW readout system for bacterial sensors, which offers significantly lower energy consumption per conversion compared to the systems described in [118,122]. This design employs an external NPN phototransistor for energy-efficient optical detection; however, this approach limits the scalability of the multi-diagnostic system. In [15], the authors developed a threshold-based bioluminescence detector that integrates CMOS photodiodes to tackle the challenges of miniaturization, while employing a dual-duty-cycling front end to achieve a low power consumption of just 59 nW. This design was fabricated using 65 nm technology reported as the first bacterial-electronic ingestible capsule for the simultaneous detection of various labile biomarkers.

#### 3.2.3. Evanescent Wave Sensors

**Figure 16:**
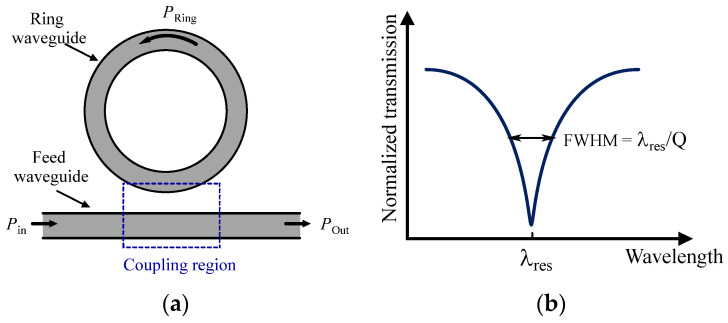
(**a**) Structure of an MRR and (**b**) Lorentzian spectrum of the ring.

Silicon photonic biosensors are among the most commonly used evanescent wave biosensors for analyses applicable to clinical diagnosis and other versatile applications, with the added benefit of scalable production through CMOS fabrication methods [124,125,126]. Silicon photonic biosensors can be categorized into two main types: resonant architecture and interferometric architecture. Each type presents its advantages and disadvantages in terms of sensitivity, limit of detection (LoD), compactness, and system complexity. A frequently employed silicon photonic structure in this field is the ring resonator [14,127,128,129,130]. For instance, the authors of [14] introduced a fully integrated electronic-photonic platform utilizing a zero-change, high-volume CMOS silicon-on-insulator (SOI) process, specifically designed for molecular and ultrasound sensing applications. By co-integrating 10 μm micro-ring resonators (MRRs) with on-chip electronics, they effectively meet the current demands for scalability, power efficiency, and compactness. This approach enables nanophotonic sensing and readout processing within a monolithic electronic–photonic system-on-chip (SoC). As depicted in Figure 16a, MRRs consist of a feed waveguide coupled to a circular waveguide. When the wavelength of the input light causes the phase shift accumulated around the ring to be an integer multiple of 2π, the ring is considered to be in resonance. At this point, destructive interference occurs at the output port of the ring, leading to maximum power circulation within the ring. Conversely, at off-resonant wavelengths, more power is transmitted to the output port, resulting in the Lorentzian-shaped transmission spectrum depicted in Figure 16b, where the full width at half maximum (FWHM) is also highlighted.

**Figure 17:**
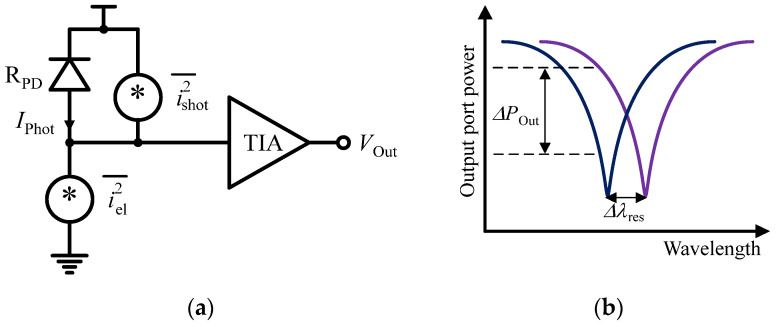
(**a**) A photodetector and a TIA transform the optical signal into the electronic domain. (**b**) The *n*_eff_ causes a resonant shift in the ring, denoted as *λ*_res_. When operating at a constant input wavelength, this resonant shift results in fluctuations in the *P*_Out_.

Resonant sensors are highly sensitive to refractive changes within the evanescent field, and they are able to detect shifts in their resonant wavelength triggered by the quantity being measured. When the refractive index near the surface of the ring resonator is altered due to the binding of target molecules, the effective index of refraction, (*n*_eff_), will also change. This variation in *n*_eff_ is detected by monitoring the resonances, which occur when the circumference *L* of the ring waveguide matches an integer multiple *m* (resonance order) of the wavelength of the feeding waveguide (as described in Equation (1)). This condition creates a negative peak in the spectrum of light exiting the ring.

(1) $$m=\frac{n_{eff}\;L}{\lambda_{res}}$$

where *λ*_res_ is the resonant wavelength. Changes in *n*_eff_ lead to shifts in *λ*_res_ [14], and this wavelength shift serves as a quantitative measure of the binding events occurring near the surface. As illustrated in Figure 17, this power fluctuation can be converted into the electrical domain using a photodetector, followed by a TIA [14].

## 4. Conclusions and Future Remarks

In this paper, the recent advances in CMOS magnetic and optical sensing mechanisms are reviewed. Examples of magnetic sensors include Hall effect, inductive, GMR, and NMR ones, while examples of optical methods include fluorescence, bioluminescence, and evanescent waves. These sensors have demonstrated significant advantages for both biological laboratories and PoC diagnostic applications. The examples discussed illustrate the breadth of innovations in this field, from sophisticated optical methods that enable high-resolution imaging and precise measurements to advanced magnetic sensors that facilitate the detection of biomarkers suing magnetic labels. These CMOS-based sensors have proven to be invaluable tools, particularly in applications where miniaturization, cost-effectiveness, and integration capabilities are paramount. As the field continues to evolve, the potential for further miniaturization and integration of these sensors with advanced data processing techniques, such as AI and ML, is immense. This integration could lead to even more powerful diagnostic tools that offer real-time analysis and greater accessibility for personalized medicine. Moreover, the scalability of CMOS technology suggests that these innovations could be mass-produced, making cutting-edge diagnostic technologies available to a broader population. In conclusion, the advancements in CMOS optical and magnetic sensors represent a significant leap forward in biosensing technology, with far-reaching implications for both research and clinical practice. As these technologies continue to mature, they are poised to revolutionize the way of diagnostics and healthcare, making them more efficient, accurate, and accessible to all.
